# Development of an enhanced recovery after surgery program for pediatric solid tumors

**DOI:** 10.3389/fsurg.2024.1393857

**Published:** 2024-05-22

**Authors:** Sara A. Mansfield, Meera Kotagal, Stephen Hartman, Andrew J. Murphy, Andrew M. Davidoff, Doralina L. Anghelescu, Marc Mecoli, Nicholas Cost, Brady Hogan, Kyle O. Rove

**Affiliations:** ^1^ Department of Surgery, St. Jude Children’s Research Hospital, Memphis, TN, United States; ^2^ Department of Pediatric Surgery, Nationwide Children’s Hospital, Columbus, OH, United States; ^3^ Department of Pediatric Surgery, Cincinnati Children’s Hospital Medical Center, Cincinnati, OH, United States; ^4^ Division of Anesthesiology, Department of Pediatric Medicine, St. Jude Children’s Research Hospital, Memphis, TN, United States; ^5^ Division of Anesthesiology, Cincinnati Children’s Hospital Medical Center, Cincinnati, OH, United States; ^6^ Department of Urology, Children’s Hospital Colorado, Aurora, CO, United States

**Keywords:** pediatric oncology, enhanced recovery after surgery, enhance recovery pathways, pediatric surgical oncology, solid tumors

## Abstract

**Introduction:**

Enhanced recovery after surgery (ERAS) is an evidence-based, multi-modal approach to decrease surgical stress, expedite recovery, and improve postoperative outcomes. ERAS is increasingly being utilized in pediatric surgery. Its applicability to pediatric patients undergoing abdominal tumor resections remains unknown.

**Methods and Analysis:**

A group of key stakeholders adopted ERAS principles and developed a protocol suitable for the variable complexity of pediatric abdominal solid tumor resections. A multi-center, prospective, propensity-matched case control study was then developed to evaluate the feasibility of the protocol. A pilot-phase was utilized prior to enrollment of all patients older than one month of age undergoing any abdominal, retroperitoneal, or pelvic tumor resections. The primary outcome was 90-day complications per patient. Additional secondary outcomes included: ERAS protocol adherence, length of stay, time to administration of adjuvant chemotherapy, readmissions, reoperations, emergency room visits, pain scores, opioid usage, and differences in Quality of Recovery 9 scores.

**Ethics and Dissemination:**

Institutional review board approval was obtained at all participating centers. Informed consent was obtained from each participating patient. The results of this study will be presented at pertinent society meetings and published in peer-reviewed journals. We expect the results will inform peri-operative care for pediatric surgical oncology patients and provide guidance on initiation of ERAS programs. We anticipate this study will take four years to meet accrual targets and complete follow-up.

**Trial Registration Number:**

NCT04344899.

## 1 Introduction

Enhanced recovery after surgery (ERAS) pathways are multi-disciplinary and multi-modal approaches to minimizing surgical stress and expediting patients' recovery after surgical procedures. ERAS was first reported by Henrik Kehlet in 1997 (1). He and his colleagues created a comprehensive care pathway for patients undergoing colonic resections. The general tenets of ERAS include limiting peri-operative fasting, maximizing opioid-sparing analgesia, early post-operative mobilization, minimally invasive techniques, and minimizing use of tubes/drains (2). This pathway allowed patients to by discharged after 2 days, compared to the previous average length of stay (LOS) of 10 days. Subsequently, this group reported that this pathway not only expedited recovery, but also decreased complications (3). ERAS ultimately became the standard of care for adult colorectal procedures (4).

Based on the improved outcomes in colorectal surgery, other surgical disciplines followed suit. The pediatric surgery community started exploring ERAS in the late 2010s. Understanding that adult principles may not be appropriate for children, some modifications were needed (5, 6). In 2018, Short et al. surveyed the American Pediatric Surgical Association's membership regarding ERAS components. Over 90% of respondents were willing to implement or were already implementing 12 of the 21 ERAS components (7). The remaining components were evaluated using a modified Delphi process, further refining general pediatric principles further (8). Since these initial studies, pediatric ERAS pathways have grown and expanded beyond colorectal procedures (9).

Given the complexity, risk (10, 11), and heterogeneity of pediatric surgical oncology procedures, ERAS has not been widely studied or adopted for this population. However, it is gaining interest (12). Here we describe the protocol used in a multi-center, prospective study: The Pediatric Oncology Recovery after Tumor Surgery (PORTS) study. The goals of the PORTS study were to determine if the proposed protocol was optimal to maximize recovery from surgery while minimizing morbidity; to characterize protocol adherence by providers and study sites; and to define short-term outcomes (90 days) from surgery after application of the ERAS protocol. We aimed to broaden exposure of the pediatric surgery community to ERAS and demonstrate its application to a variety of procedures and institutions.

## 2 Methods and analysis

### 2.1 Protocol development

Adult and existing pediatric ERAS protocols were reviewed. The Pediatric Urology Recovery After Surgery Endeavor (PURSUE) trial formed the basis of the protocol, given its success (13) and similarities in surgical complexity to the pediatric oncology patient population (14). The study team determined protocol inclusion criteria based on literature review, observed issues in pediatric patients undergoing solid tumor resections, and group consensus.

**Figure 1:**
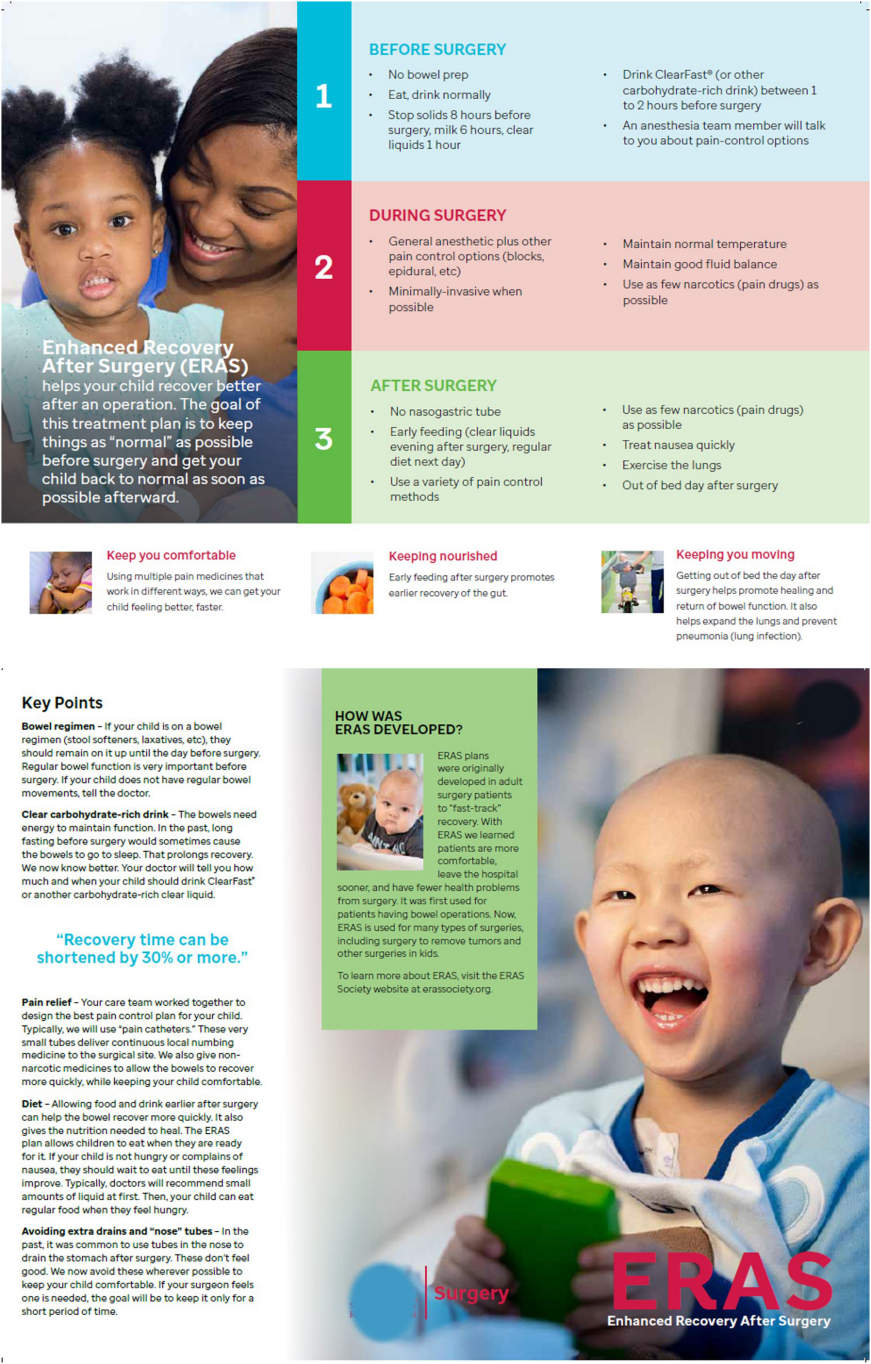
Example of patient education materials for the pediatric oncology recovery after tumor surgery (PORTS) study.

Managing expectations of patients and families was considered critically important to the success of the protocol. Each center was tasked with developing educational materials to explain the rationale for each process measure and provide clear criteria for discharge. An example of one such brochure is provided in Figure 1. Centers were encouraged to review the educational tools as part of the pre-operative clinic visit.

A key tenet of ERAS protocols is reduction in opioid consumption peri-operatively. Opioids are associated with sedation and slower return of bowel function. In addition, survivors of childhood cancer have higher risks for substance use disorders (15). Therefore, the protocol focused on opioid-sparing analgesia intra-operatively and post-operatively. Regional analgesia was individualized to each patient, but ideally administered prior to the case. Epidural analgesia was preferred for larger laparotomies. Regional blocks were used for smaller incisions, or in cases of parental request, or contraindications to epidural catheter placement. With regional analgesia instituted pre-incision (i.e., epidural catheter placed and bolus dose administered, for open procedures), the intra-operative opioid needs are expected to be lower. The intraoperative goal for this study was ≤0.3 mg/kg IV milligram morphine equivalents (MME). This goal was based on a prior pediatric urology pilot study (13). During implementation, it was emphasized that patient-specific factors can and should override this opioid-sparing goal when needed.

Centers were encouraged to utilize minimally invasive techniques for surgery whenever feasible. There are numerous benefits to minimally invasive surgery (MIS) including decreased pain with less opioid need and shorter LOS (16). While MIS is gaining momentum in pediatric surgical oncology (17), it is still not universally applicable. While MIS utilization was encouraged, oncologic principles were emphasized as the utmost priority to obtain tumor-specific operative standards (18). If laparoscopic or robotic techniques were utilized for any portion of the operation, this was considered to meet the process measure. The degree of MIS utilization was tracked.

Maintaining normothermia between 36°C and 38°C during the operative period was included in the protocol. Any value outside this range would nullify this measure. There is evidence in adults that normothermia decreases infectious complications. This also impacts metabolic demands (19), stress responses (20), and pharmacokinetics of anesthetics (21). Methods for ensuring normothermia were individualized to each center. We recommended pre-warming the room and having at least one warming device in place during the case.

Malignancy is often considered a risk-factor for deep vein thrombosis (DVT) in adults. Therefore, this protocol included the use of sequential compression devices prior to induction of anesthesia for all patients older than 10 years. Pharmacologic intervention was left up to individual teams as there is no data to support its routine use in children. Recent studies have questioned whether there is truly an increased risk of DVT for children with solid tumors (22). This process measure was kept in the protocol and tracking of its impact will be assessed.

An additional tenet of ERAS protocols is ensuring euvolemia. This can be challenging in a pediatric protocol in which patients have a wide range of weights, age, and complexity of operations. We set a goal of 3–7 ml/kg/h of operating time, for crystalloid and colloid fluids, based on prior benchmarks (13). Additional volume of blood products could be given for blood loss as needed. Use of cardiac output and volume monitoring adjuncts were encouraged based on availability at each center. Additionally, bowel preparation was discouraged, as this impacts the pre-operative volume status by causing dehydration. Post-operatively, IV fluids were stopped as soon as the patient demonstrated tolerance of oral fluids, ideally on post-operative day (POD) 1. A minimal IV rate was allowed to “keep the vein open” (usually 20 ml/h or less).

Post-operatively, acetaminophen and ketorolac or ibuprofen were given as scheduled dosing, unless there were contraindications. The use of these medications may often be a concern in pediatric oncology patients. However, for patients receiving neoadjuvant chemotherapy, at the time of tumor resection, cell counts have or are recovering, and neutropenia and/or thrombocytopenia are not present. There has not been any evidence to suggest these medications to be unsafe perioperatively in these patients. Historically, there were concerns about post-operative bleeding in patients given non-steroidal anti-inflammatories drugs (NSAIDs). However, this has not been supported in modern studies (23–25). Additionally, there does not appear to be an increased risk of renal dysfunction in children with normal renal function pre-operatively. Specifically, children with Wilms tumor likely do not have an abrupt decrease in glomerular filtration rate (GFR) following nephrectomy as the contralateral kidney likely functionally adapts prior to surgery. Therefore, the protocol encouraged scheduled ibuprofen or ketorolac postoperatively, even in patients undergoing nephrectomy with normal creatinine or cystatin c pre-operatively. This study collected details regarding post-operative kidney function to analyze the impact of NSAIDs in these patients.

We also aimed to decrease opioid consumption post-operatively. In the setting of multi-modal pain control and regional analgesia, many patients may require minimal to no opioids post-operatively. The goal of this study was opioid consumption ≤0.15 mg/kg/day IV MME per post-operative day. This goal came from a prior benchmark study which found that 75% of patients could safely achieve this goal without an increase in pain (13). We recommended ordering doses of 0.05 mg/kg IV MME every 4–6 h as needed, with instructions to call if administration of three doses in a block of 24 h appeared to be insufficient, to troubleshoot the pain regimen. It was noted that pre-implementation of the ERAS protocol, opioids may have been administered for a range of complaints (IV site pain, back pain from lying in bed, etc.). During the implementation period, non-opioid options were emphasized; furthermore, in the immediate postoperative period, non-opioid analgesia was administered as scheduled doses with acetaminophen (initially IV, followed by PO) and ketorolac IV, followed by ibuprofen PO. After the first post-operative day, the scheduled doses could be switched to as needed (PRN) based on patients' pain with specific ranges of pain intensity, to encourage acetaminophen and/or ketorolac use prior to opioids.

To improve peri-operative nutrition, all patients were allowed to continue their baseline diets until 8 h prior to operation (solids), with clear liquids allowed until 1–2 h pre-operatively. Breast milk and formula recommendations were also encouraged up to each institution's required nil per os (NPO) time point. Additionally, carbohydrate-rich clear beverages such as ClearFast, RecoverAid, Gatorade, or Pedialyte were encouraged the morning of surgery (either 1 or 2 h pre-operatively depending on institution's NPO guidelines). Orogastric (OG) or nasogastric (NG) decompression was utilized during the operation as needed, but OG/NG tubes were removed prior to leaving the operating room unless there was a specific indication to maintain them *in situ*. We aimed to allow patients clear liquids following recovery of anesthesia and advance to baseline diet as tolerated, with a goal of starting a regular diet by POD#1. Antiemetics were ordered PRN for each patient. Surgeon discretion was allowed based on type of operation. If patients were kept NPO longer due to concern of proximal gastrointestinal track procedure, they were allowed to stay on study. Post-operative bowel function, vomiting, and need for NG insertion were tracked as part data collection.

To prevent deconditioning, patients were to get out of bed by POD#1. Transferring to a chair would qualify, whereas sitting up in bed would not. Centers were encouraged to include physical therapy for these patients. Surgical drains, urinary catheters and NG tubes likely impact patient comfort and willingness to ambulate. Therefore, these were discouraged where feasible. For example, urinary catheters were used in the setting of lumbar epidural analgesia to prevent urinary retention. Additionally, sometimes surgical drains were necessary based on the operation. To account for those patients in whom a clinical decision has been made to leave an intraperitoneal drain, a postoperative measure was added that any such drains should be removed on or by POD#3. This day was proposed based on pilot data showing that many patients are ready to go home and that these drains are rarely helpful.

A full list of the 20 ERAS process measures is included in Table 1. This protocol was adopted by all centers with definitions of measures kept uniform between sites.

**Table 1:** Protocol measures—pediatric oncology recovery after tumor surgery (PORTS) study.

	Process measure	Goal
Preoperative	Counsel about ERAS	Set expectations via handout + pre-operative checklist either by phone or in clinic
	Carbohydrate load	10 ml/kg (up to 350 ml) ClearFast or RecoverAid (alt: Gatorade, PowerAde, Pedialyte) between 1 and 2 h scheduled start time. Omit if <6 months age.
	Avoid prolonged fasting	Regular diet night before, no prolonged clear liquid diet
	No bowel preparation	
	Antibiotic prophylaxis	Case-appropriate within 60 min prior to incision
Intraoperative	Regional anesthesia	Pre-incision epidural, TAP, QL, or ESP catheters preferred
	Avoiding excess drains	No intraperitoneal or subcutaneous drains
	Euvolemia	3–7 ml/kr/h crystalloid and colloid fluids (over OR time)
	Normothermia	36°C–38°C during skin-to-skin time
	Minimizing Opioids	≤0.3 mg/kg IV milligram morphine equivalents
	DVT prophylaxis	SCDs prior to induction for patients ≥10 years
	Minimally invasive approach	Laparoscopic or robotic as oncologically feasible
Postoperative	No nasogastric tube	Remove prior to leaving OR if used
	Nausea/vomiting prophylaxis	PRN ondansetron, diphenhydramine, scopolamine, etc.
	Early feeding	Clears on POD#0, regular diet by POD#1
	Early mobilization	Out of bed by POD#1
	Postoperative non-opioid pain regimen	Scheduled acetaminophen and NSAID (ketorolac, ibuprofen); Avoid NSAID if CKD 2+, allergy
	Early removal of IV fluids	By POD#1
	Early removal of drains	As early as possible, Foley removal at time of epidural removal, if applicable
	Minimizing opioids	≤0.15 mg/kg IV milligram morphine equivalents per day

POD, post-operative day; NSAID, non-steroidal anti-inflammatory drugs; CKD, chronic kidney disease; TAP, transversus abdominus plane; QL, quadratus lumborum; ESP, erector spinae plane.

### 2.2 Study design and objectives

This was a prospective case control study. Retrospective historical controls included patients undergoing similar operations at each center in the five years prior. These patients were propensity-matched by confounding variables, including: age, sex, prior surgery, and other comorbidities such as immunosuppression, chronic kidney disease (CKD), and weight/height/BMI. All patients older than one month undergoing abdominal, pelvic, or retroperitoneal tumor resections were included. The study centers were all free-standing children's hospitals. The primary outcome was 90-day complications per patient (listed in Table 2). Secondary outcomes included: ERAS protocol adherence, LOS or time to transfer to the oncology service, time to chemotherapy, time to clearance for chemotherapy, postoperative emergency room (ER) visits, readmissions, reoperations, pain scores, and opioid usage. Inpatient data points were collected for each post-operative day up to seven. Oncologic details regarding tumor type, stage, and outcomes were also included.

**Table 2:** List of short-term complications by Clavien-Dindo classification.

**Grade I complications**	**Grade II complications**
Electrolyte disturbance	Blood transfusion
Fever (≥38°C)	Ileus requiring NG tube ± TPN + nausea/vomiting
IV complication (infiltration)	Infection/bacteremia treated with Abx ± fever
Nausea/vomiting	Infection/pyelonephritis treated with Abx ± fever
Neuropraxia (positioning complication)	Infection/superficial wound treated with bedside drainage, Abx ± fever
trAnsient elevation in serum creatinine	Infection/UTI treated with Abx ± fever
Wound dehiscence	Infection/GI infection with Abx ± fever ± diarrhea
incisional seroma	Urinary retention requiring catheterization or indwelling urinary catheter
Other grade I	Venous thromboembolism
**Grade III complications**	Lymphocele or chylous ascites treated conservatively with diet changes
Abdominal abscess requiring IR/OR drainage, Abx	Other grade II
Bowel leak treated surgically in OR	**Grade IV complications**
Catheter malfunction/loss requiring placement in OR	Respiratory failure requiring ventilation, ICU
Fascial dehiscence/evisceration treated in OR	Renal failure, ICU
Hemorrhage requiring embolization or OR	Multiorgan failure, ICU
Small bowel obstruction treated surgically in OR	Sepsis, septic shock, ICU
Bile or pancreatic leak requiring IR or OR drainage	Other grade IV
Urinoma requiring IR/OR drainage	
Ureteral obstruction requiring intervention	**Grade V complications**
Lymphocele or chyle leak requiring IR or OR intervention	Death

TPN, total parenteral nutrition; NG, nasogastric; abx, antibiotic; UTI, urinary tract infection; IR, interventional radiology; OR, operating room; ICU, intensive care unit.

The study was originally powered to require 259 historical controls and 129 ERAS patients (2:1 matching ratio). With 80% power, we would be able to detect a 15% absolute reduction in complications with application of the ERAS protocol. After examining the available historical controls and observing an increased baseline proportion of patients experiencing any postoperative complication, we were only able to accommodate a 1:1 ratio match with available controls and prospective enrollment was stopped after 95 patients.

All enrolled study patients and families received a pre-operative questionnaire (to be completed between 90 days prior to surgery up to the pre-operative area). A Modified Quality of Recovery 9 (QoR-9) questionnaire was provided once during the hospitalization (POD#1–14). Post-operative questionnaires are to be filled out at the first scheduled surgery clinic visit after discharge from the hospital (POD#15–90). There were additional separate, short questionnaires for both patients and parents/guardians regarding time off work and/or school.

### 2.3 Implementation

IRB approval was obtained at each individual institution. Principal investigators at each center identified key stakeholders in the surgery, anesthesia, physical therapy, nutrition, peri-operative nursing, outpatient clinic nursing, inpatient nursing, and oncology teams. Patient education brochures were created in coordination with literacy experts at each institution (Figure 1). The protocol and study objectives were discussed with all parties. The same protocol was utilized at each center. After collaborators buy-in and IRB approval, the study was started.

Surveys were administered to key personnel within the surgery and anesthesia teams to understand baseline knowledge of ERAS and perceived barriers to implementation. This was used to guide implementation and overcome barriers. This allowed investigators to focus education on perceived issues or concerns with ERAS pathways. Investigators were required to present the protocol, rationale, and supporting evidence multiple times to every team involved in this study. Identifying and engaging key stakeholders was found to be critically important to keep the study on track. Reaching adherence to all ERAS protocol items, though desirable, was unlikely to be achieved in all study participants. Clinical judgement was encouraged to take precedence over following the ERAS measures strictly, expecting that some patients will have clinical and circumstantial reasons for not meeting a particular protocol item measure. Non-adherence to one or more protocol items did not disqualify patients from the study, particularly since the study was designed to be observational and collect data about standard of care only.

**Figure 2:**
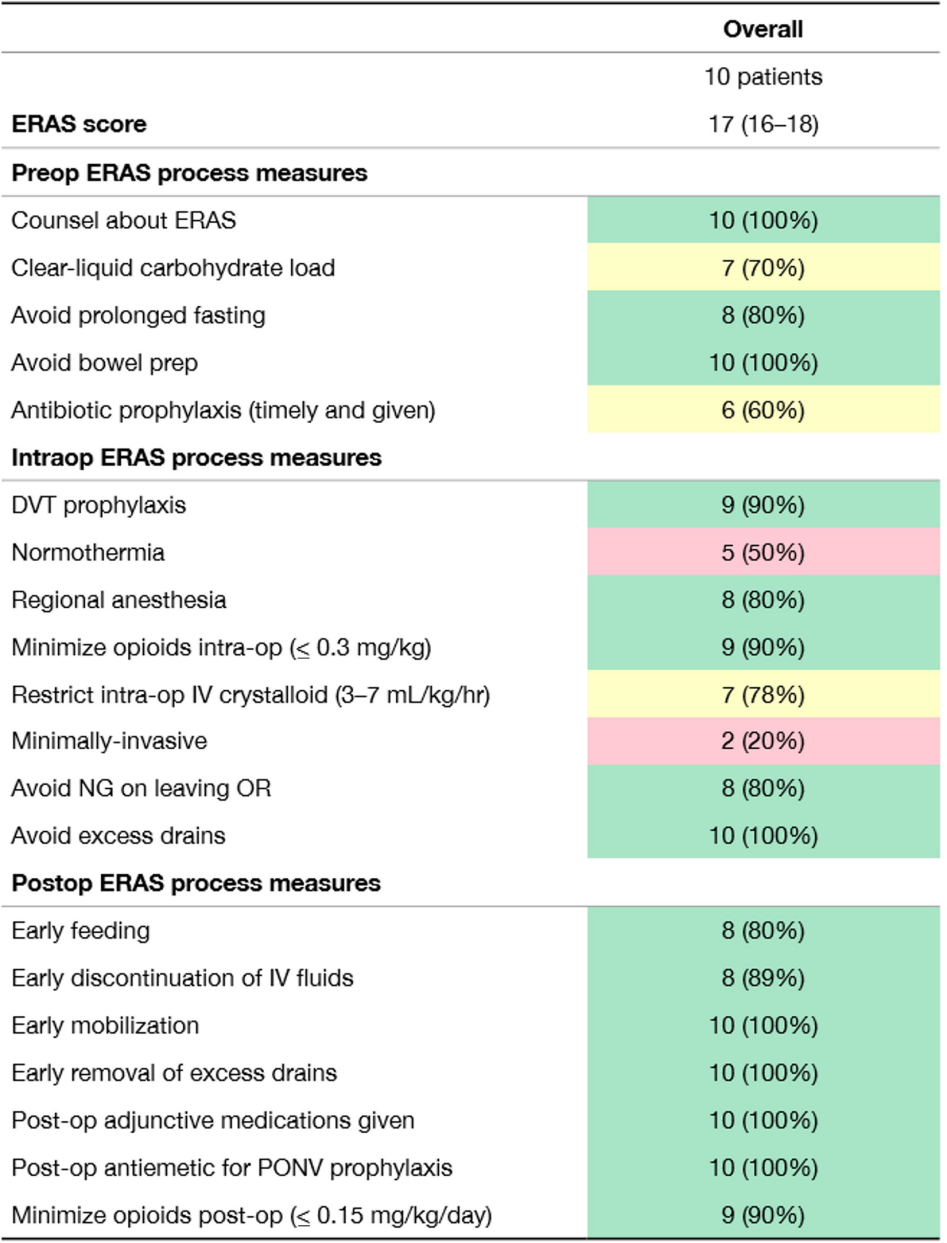
Sample report card demonstrating process measure adherence through the first 10 patients. ERAS score represents median number of process measures achieved per patient.

To verify integrity of the ERAS implementation at each site, an *a priori*-designated audit committee evaluated all the ERAS care principles for the first 10 study participants at each institution and additionally at any time a site investigator wished to have one. The purpose of the audit committee call was to discuss ongoing ERAS compliance based on audit data in the Research Electronic Data Capture (REDcap) database, with the aim of further identifying barriers to implementation and problem areas with pre-, intra-, and post-operative ERAS care principles and ideas of how to strengthen their use consistently in all enrolled study patients. An example of an audit report card is provided in Figure 2. These were used to focus on areas for improvement.

### 2.4 Data collection

Regardless of the number of process measures followed according to the protocol, each enrolled patient continued on the study with prospective data collection in REDcap. Data points included oncologic and surgical details, pre-operative co-morbidities, prior operations, chemotherapy, and radiation. Intraoperative fluids (type and volume), opioids (type and doses), operating room time, regional analgesia details, temperature, and complications were collected. For each post-operative day, patient weight, fluid status (intake and output), opioid consumption, pain scores, mobility, diet, serum creatinine, and complications were recorded. Length of stay or days until transfer to the oncology service for chemotherapy were recorded. Time to clearance for adjuvant cancer therapies and start dates were recorded.

Where possible, REDcap prompts were inserted to provide additional documentation when process measures were not met. For example: if a patient had a NG tube left in place post-operatively, this would be captured. A text box would then prompt a description of why one was left in place. While we may not be able to determine exact cause of non-compliance, we hope that comprehensive data collection will aid in determining the feasibility of this protocol.

Patients had post-discharge follow-up surgery clinic visits according to surgeon/institutional preference. Study investigators prospectively inquired about any ER visits out to 90 days from surgery (including ERs outside of study-site system), unexpected returns to the operating room, readmissions, and noted any complications. The Clavien-Dindo classification for complications was utilized (26). Patients that had both higher-grade and a lower-grade complications were only coded according to the higher-grade complication, not both (e.g., ileus requiring NG tube with nausea/vomiting is a grade II complication; such a complication should not also be coded with grade I—nausea/vomiting).

### 2.5 Historic controls

Baseline data on all historical controls who meet study inclusion criteria as above for a period of five years prior to the study start were collected. Data points used for propensity-matching included: age, sex, diagnosis, type of operation performed, mobility status, history of prior abdominal surgery, CKD status, presence of immunocompromise, history of radiation, or history of chemotherapy. Matched patients then underwent retrospective chart review for extraction of the same information gathered for the prospectively-enrolled cohort.

## 3 Discussion

Here we have described the implementation strategy for our multi-center, prospective Pediatric Oncology Recovery after Tumor Surgery (PORTS) study. Results of this study will inform expansion of ERAS principles to pediatric patients undergoing solid tumor resections. One can easily see how this patient population would benefit from ERAS principles. Patients frequently experience nutritional alterations due to tumor-related cachexia and cancer-directed therapy. Deconditioning related to therapy and fatigue are also common. Patients also are exposed to opioids for tumor-related pain, treatment side-effects, or secondary to painful procedures. Worsening of any of these aspects peri-operatively has the potential to decrease their performance status further and potentially impact their ability to start and tolerate adjuvant cancer-direct therapies.

This protocol is unique in that it will be utilized for a variety of solid tumor resections. Moon et al. reported on a single-institution experience of ERAS for Wilms tumor resections (25). While the methodology and exact protocol are different, this study supports early feeding and routine use of ketorolac following nephrectomy. Notably nephrectomy for Wilms tumor carries a low complication rate at 0.5% (27) and may not have the same complexity as other abdominal tumor resections. Zhu et al. reported on ERAS use in localized retroperitoneal neuroblastomas (28). However, this protocol still utilized post-operative nasogastric tubes and had a median length of stay of 9 days. They did demonstrate that lower intraoperative fluid volumes were achievable without worsening complication rates. Whether our ERAS protocol can be applied to more complex operations remains unclear and provides impetus for the proposed study. The pragmatic design may allow us to evaluate which process measures are most impactful to recovery of our patients.

The protocol was developed to be applicable to a wide range of operations and resections. However, the recovery challenges and possible complications for each procedure are unique. Pending the results of this protocol, the pathway should be fine-tuned for individual procedures. For example, patients undergoing extensive hepatectomy may have a risk of coagulopathy post-operatively. This impacts the ability to safely remove the epidural catheter due to risk of an epidural hematoma (29, 30). Therefore, further evaluating and fine-tuning regional analgesia for these cases warrants further evaluation in future protocols (31).

The PORTS study will be the first multicenter, prospective, propensity-matched, case–control cohort study to evaluate ERAS in pediatric surgical oncology patients. This protocol marks the first phase of a collaborative quality improvement effort within the pediatric surgical oncology community to improve and standardize care of oncology patients. Results of the study will allow further refinements with the ultimate goal to improve patient outcomes and readiness for adjuvant cancer-directed therapies.
